# Pathway-based discovery of genetic interactions in breast cancer

**DOI:** 10.1371/journal.pgen.1006973

**Published:** 2017-09-28

**Authors:** Wen Wang, Zack Z. Xu, Michael Costanzo, Charles Boone, Carol A. Lange, Chad L. Myers

**Affiliations:** 1 Department of Computer Science and Engineering, University of Minnesota, Minneapolis, MN, United States of America; 2 HealthPartners Institute, Minneapolis, MN, United States of America; 3 Donnelly Centre, University of Toronto, Toronto, ON, Canada; 4 Departments of Medicine and Pharmacology, Masonic Cancer Center, University of Minnesota, Minneapolis, MN, United States of America; Case Western Reserve University School of Medicine, UNITED STATES

## Abstract

Breast cancer is the second largest cause of cancer death among U.S. women and the leading cause of cancer death among women worldwide. Genome-wide association studies (GWAS) have identified several genetic variants associated with susceptibility to breast cancer, but these still explain less than half of the estimated genetic contribution to the disease. Combinations of variants (i.e. genetic interactions) may play an important role in breast cancer susceptibility. However, due to a lack of statistical power, the current tests for genetic interactions from GWAS data mainly leverage prior knowledge to focus on small sets of genes or SNPs that are known to have an association with breast cancer. Thus, many genetic interactions, particularly among novel variants, remain understudied. Reverse-genetic interaction screens in model organisms have shown that genetic interactions frequently cluster into highly structured motifs, where members of the same pathway share similar patterns of genetic interactions. Based on this key observation, we recently developed a method called BridGE to search for such structured motifs in genetic networks derived from GWAS studies and identify pathway-level genetic interactions in human populations. We applied BridGE to six independent breast cancer cohorts and identified significant pathway-level interactions in five cohorts. Joint analysis across all five cohorts revealed a high confidence consensus set of genetic interactions with support in multiple cohorts. The discovered interactions implicated the glutathione conjugation, vitamin D receptor, purine metabolism, mitotic prometaphase, and steroid hormone biosynthesis pathways as major modifiers of breast cancer risk. Notably, while many of the pathways identified by BridGE show clear relevance to breast cancer, variants in these pathways had not been previously discovered by traditional single variant association tests, or single pathway enrichment analysis that does not consider SNP-SNP interactions.

## Author summary

Susceptibility to breast cancer is partially encoded in our genomes, but despite the development of new genomic technologies over the past decade, we are still not able to accurately predict disease susceptibility from genome sequences. One reason for this gap is that we lack methods for finding combinations of genome variants that lead to disease. Extensive studies in model organisms have experimentally constructed millions of double mutants to study genetic interactions and have defined the basic principles by which genes combine to cause phenotypes in an organism. One powerful outcome of these studies in model systems is that genetic interactions frequently form highly organized patterns that can be used as a basis for improved detection of them in humans. We developed a novel computational approach based on this principle for identifying pathway-level interactions that contribute to breast cancer disease risk. Applying this method to six different groups of breast cancer patients, we identified a core set of pathways, including glutathione conjugation, vitamin D receptor, purine metabolism, mitotic prometaphase, and steroid hormone biosynthesis. These pathways are well-supported across multiple cohorts and may contribute to breast cancer susceptibility.

## Introduction

Cancer, like many common diseases, is influenced by a variety of genetic and environmental factors. With the rise of inexpensive genotyping technologies, the portion of risk due to inherited genetic variants has been measured with unprecedented resolution. A recent comprehensive study reported excess familial risk for 20 of 23 cancer types with an overall heritability estimate of 33% [1]. This varied across different cancer types, from prostate cancer and breast cancer on the high end with estimated heritabilities of 57% and 31% respectively, to head and neck cancers on the low end with an estimated heritability of 9% [1, 2]. This study concluded that for most cancers, our risk is at least partially influenced by the genes we inherit.

As with other heritable diseases, there has been substantial interest in identifying specific genetic loci that increase or decrease an individual’s risk for specific cancers. Over the past decade, genome-wide association studies have been the primary strategy for discovering such loci, and indeed, have been successful at identifying a large number of single-nucleotide polymorphisms (SNPs) with statistically significant association to a variety of diseases including cancer [3–7]. However, for most diseases, there remains a large disparity between the disease risk explained by the discovered loci and the estimated total heritable disease risk based on familial aggregation [8–13]. For example, for breast cancer, there have been approximately 100 risk loci identified to date through genome-wide association studies, but the combination of these loci explains only approximately one-third of the genetic contribution to breast cancer risk [1], a scenario that is typical across many diseases. There are a variety of explanations for this phenomenon, commonly referred to as “missing heritability.” For example, one explanation is that disease risk is modulated by a large number of loci, each having a relatively small effect [8–12, 14]. Alternatively, it has been proposed that rare variants, which are not measured by most microarray-based genotyping platforms, may be responsible [8–12, 14]. Yet another possible explanation for our inability to explain the genetic component of disease is genetic interactions between combinations of common and/or rare loci [10, 11, 13, 15, 16].

Genetic interactions describe combinations of two or more genetic variants whose combined contribution to a phenotype cannot be explained by their independent effects [13, 17, 18]. In principle, genetic interactions can also be discovered through genome-wide association analysis by measuring the associations between specific combinations of variants and the disease phenotype. However, in practice, the large number of possible combinations introduces both computational and fundamental statistical challenges. For a typical genotyping array, computing associations for all possible pairs (e.g. 10^11^ for 500k SNPs) is a daunting computational task. While there have been efficient and scalable computational tools developed for this purpose [19–22], even when association tests can be computed, statistical power is too limited to support genome-wide discovery of SNP-SNP interactions [13].

**Fig 1:**
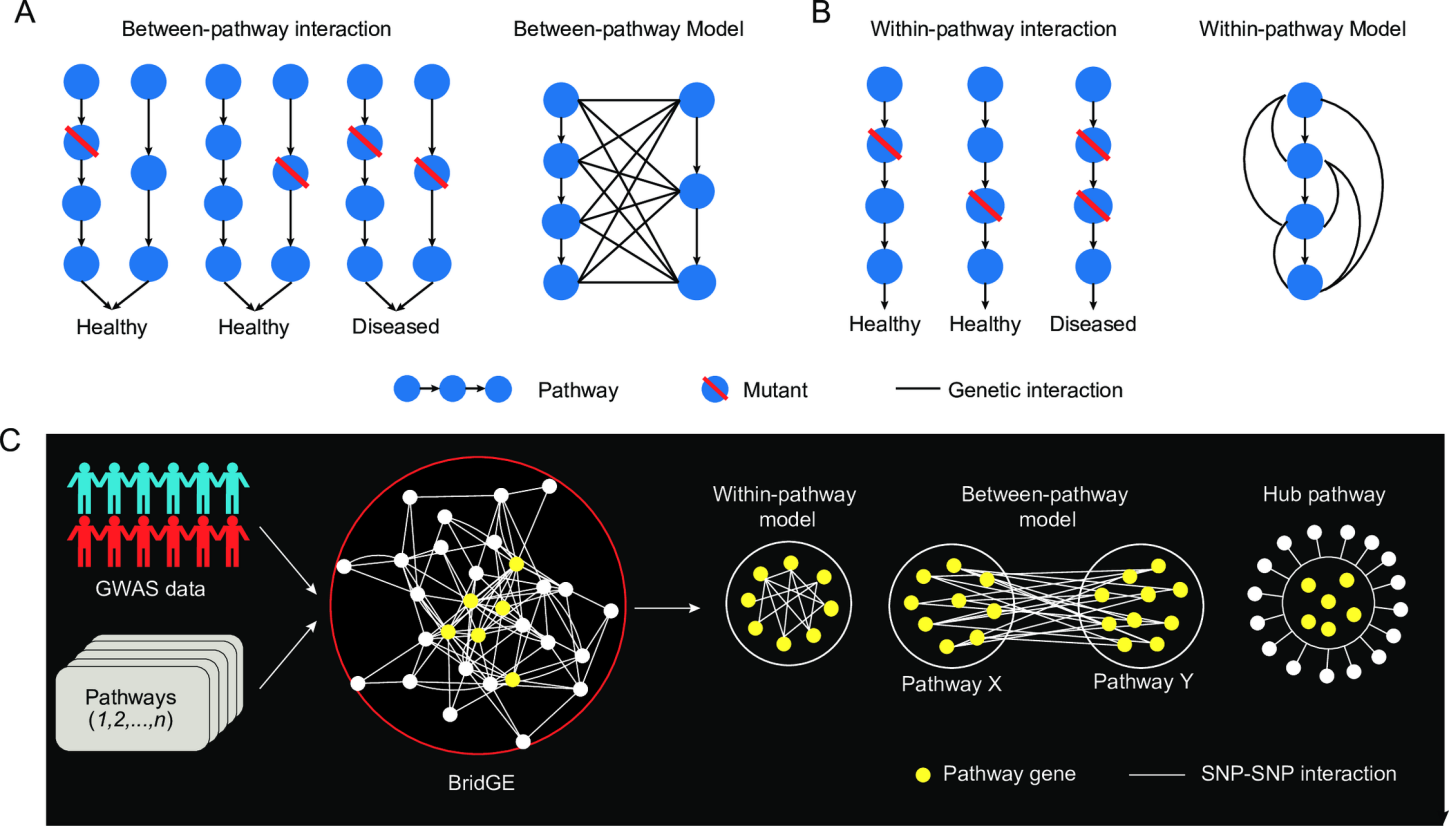
Pathway-level genetic interaction models. (A) Between-pathway interaction and between-pathway model. Two biological pathways share a common function necessary for maintaining a healthy state. Genetic variants in individual pathways do not result in a phenotype, but joint mutations in both pathways in the same individual results in disease. Between-pathway interactions clustering between two complementary pathways and appear are referred to as an instance of the between-pathway model (BPM). (B) Within-pathway interaction and within-pathway model. A single pathway supports a function for maintaining a healthy state. A single genetic variant does not result in a phenotype, but joint mutations in the same pathway results in the loss of function and a disease state. Within-pathway interactions clustered within the single pathway are called a within-pathway model (WPM). (C) Overview of the framework for discovering pathway-level genetic interactions from GWAS breast cancer data, leveraging the BridGE method [23].

We recently developed a novel method, called BridGE, for discovering genetic interactions from genome-wide association studies [23]. The approach was designed based on key insights from reverse-genetic interaction screens in model organisms where it has been observed that genetic interactions frequently cluster into highly structured motifs [24–27]. More specifically, genetic interactions often cluster into coherent groups that connect or bridge across two distinct pathways. In other words, if variants in two different genes, each belonging to a different pathway, result in a genetic interaction, then any pairwise combination of deleterious SNPs in genes annotated to the two pathways should exhibit a similar interaction phenotype. We refer to this type of genetic interaction structure as a “between-pathway” model [28]. The BridGE approach leverages this idea to explicitly search for coherent sets of SNP-SNP interactions within GWAS cohorts that connect groups of genes corresponding to characterized pathways or functional modules. Although many pairs of loci do not have statistically significant interactions when considered individually, interactions can be collectively significant if there is an enrichment of SNP-SNP interactions between two functionally related sets of genes (Fig 1A). The method imposes prior knowledge of pathway membership to exploit the expected between-pathway topology of genetic networks [23]. Because the number of hypothesis tests performed for all possible between-pathway combinations is substantially less than the number of tests for all possible SNP pairs (~10^5^ as compared to ~10^11^), this enables us to extract statistically significant pathway-level interactions that can be associated with either increased or decreased risk of disease.

In this study, we describe the application of our BridGE method to breast cancer as part of the “Up for a Challenge—Stimulating Innovation in Breast Cancer Genetic Epidemiology” (U4C) competition. Breast cancer is the second largest cause of cancer death among women in the U.S. with approximately 40,000 deaths annually [29]. GWAS studies have been quite successful at identifying a number of susceptibility loci for breast cancer in a variety of populations [30–37], but as described earlier, the known loci still explain only a limited portion (~one-third) of the measured heritability [1], suggesting that there are new genetic factors to be discovered. The U4C challenge presented a unique opportunity to apply our new method to several different breast cancer cohorts representing more than five different ethnic populations and enabled a detailed analysis of how genetic interactions vary across different patient populations.

We describe new pathway-level genetic interactions discovered across four U4C studies (six independent cohorts) (Table 1) [38–42]. Our approach can discover between-pathway interactions, as described above, as well as within-pathway interactions, which are pairwise combination of SNPs in genes annotated to the same literature-curated pathway. We also describe the identification of pathways that participate in many interactions without exhibiting a specific local structure (i.e. “hub-pathways”). Independent discoveries from each cohort are discussed along with replication analysis where the proper cohorts exist. We conclude with a consensus analysis of genetic interactions, which revealed a set of new pathways that are associated with breast cancer across multiple cohorts.

**Table 1:** Information about the 4 GWAS data sets used in this study.

Study	Accession	Platform	Case	Control	Population	Subtype	Number of Samples used in BridGE
BPC3	phs000812	Illumina HumanHap550v3.0; HumanHap 660	1998	3263	European	ER negative	3490
CGEMS	phs000147	Illumina HumanHap550	1145	1142	European	Primarily ER positive	2244
MCS	phs000517	Illumina Human660W-Quad_v1_A, Human 1M	1878	1830	Japanese, Latina, African American	Primarily ER positive	1364, 282, 390
SBCGS	phs000799	Affymetrix 6.0	2867	2285	Chinese	Mixture of ER positive/ negative	imputed 4562, non-imputed 4490

## Results and discussion

### BridGE: A method for systematic discovery of pathway-level genetic interactions

**Fig 2:**
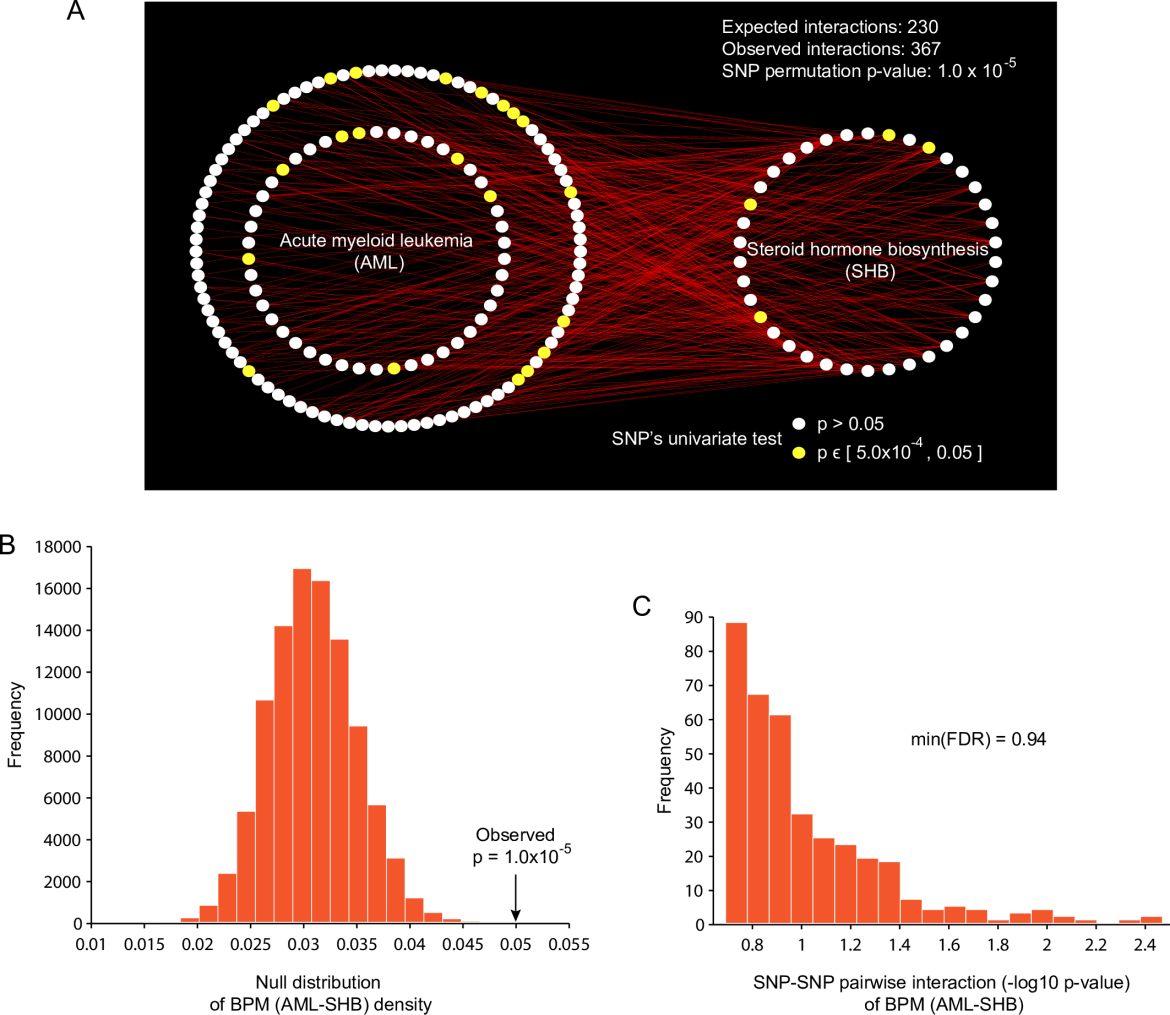
An example between pathway interaction identified from the BPC3 cohort. (A) Interaction between Acute Myeloid Leukemia (AML) gene set and Steroid hormone biosynthesis (SHB) gene set. White and yellow nodes represent the SNPs mapped to genes in the corresponding pathways and their color shows the significance of a univariate test in the same breast cancer cohort (white: not significant; yellow: marginally significant, 10^−4^ < p < 0.05). Red lines indicate the risk associated SNP-SNP interactions between SNPs mapped to the corresponding pathways. (B) Null distribution of the SNP-SNP interaction density between the AML and SHB based on 200,000 SNP permutations. The arrow indicates the observed SNP-SNP interaction density in the BPC3 cohort. (C) Distribution of the significance of pairwise SNP-SNP interactions (-log_10_ p-value) tested individually for SNP pairs supporting the AML-SHB interaction. The most significant SNP-SNP interaction results in an *FDR* = 0.94 after multiple hypothesis correction, suggesting that there is not sufficient power to detect SNP-SNP interactions between these pathways in this cohort.

**Fig 3:**
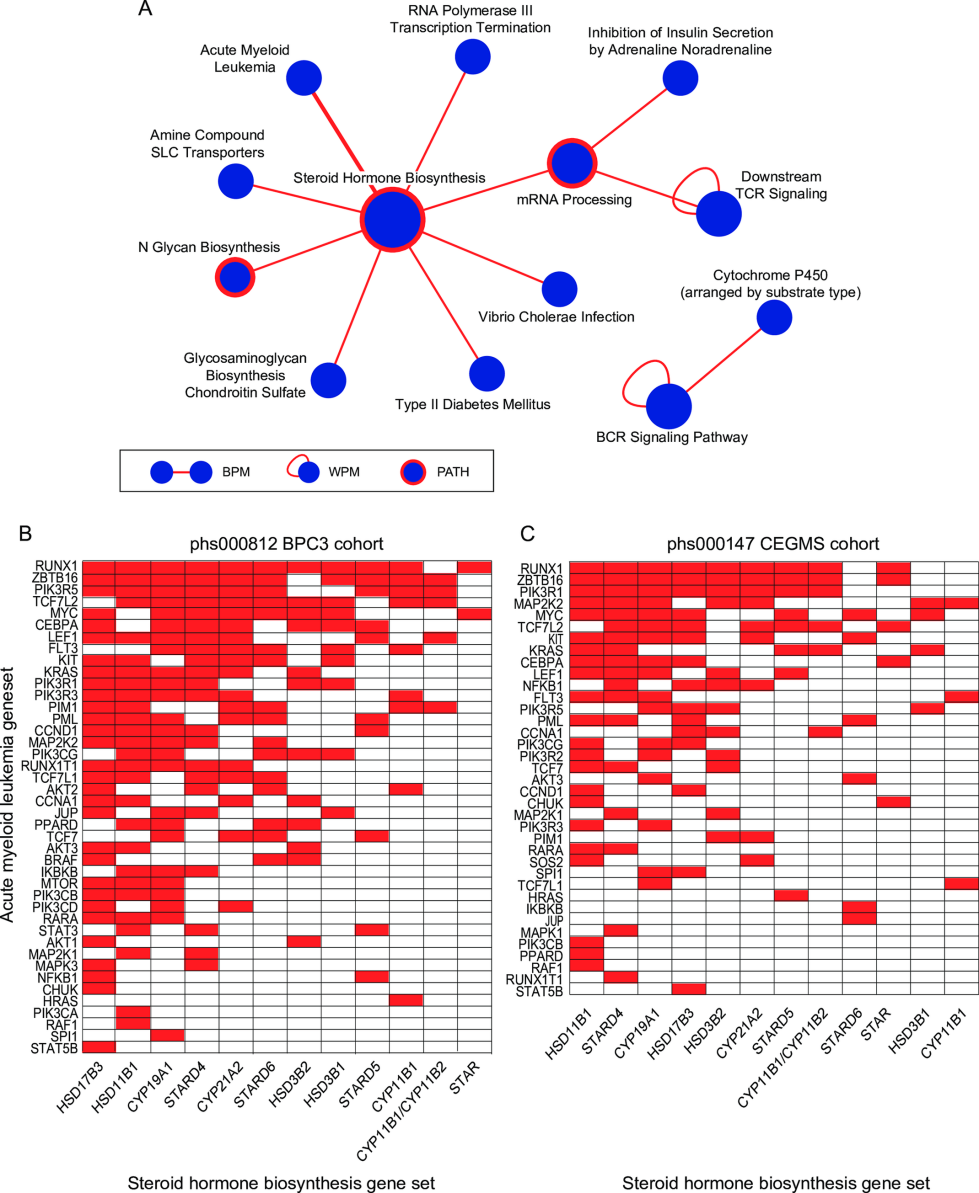
Summary of between-pathway and within-pathway interactions discovered from the phs000812 (BPC3) cohort. (A) Network representation of a set of significant (*FDR* ≤ 0.25) pathway-level interactions (BPM, WPM and PATH) that are associated with increased risk of breast cancer. Node size reflects the interaction degree. (B & C) Heatmap view of the interaction between the Acute Myeloid Leukemia (AML) gene set and the steroid hormone biosynthesis (SHB) pathway in the discovery cohort BPC3 (B) and in the replication cohort CGEMS (C). Red in the heatmap indicates that there is at least one SNP-SNP interaction identified between the corresponding genes.

We applied our recently developed method, BridGE, to explicitly search for pathway-level genetic interactions from genome-wide association study (GWAS) data [23]. The details of our method are described in our companion paper [23], but a brief overview is provided as part of this study (Methods). In general, BridGE takes as input human genotypes from matched disease/control groups, typical of that used for GWAS, together with a set of pathways as defined by curated functional standards (e.g. KEGG[43], Reactome [44], Biocarta[45]). The method then searches for instances of three different pathway-level models of genetic interactions, all motivated by analysis of genetic interactions in yeast [24–27, 46]: (1) between-pathway model (BPM) (Fig 1A and 1C), (2) within-pathway model (WPM) (Fig 1B and 1C), and (3) hub pathways (PATH) (Fig 1C). Between-pathway interactions occur when two pathways impinge on a common function required to maintain a healthy (non-disease) state. Because the two pathways can functionally compensate for each other, the disease phenotype only occurs when both pathways are perturbed in the same individual. Under the within-pathway model, a single genetic variant partially disables a pathway’s function but, when combined with another deleterious variant affecting the same pathway, complete loss of pathway function results and leads to a disease state. Pathway hubs correspond to pathways with frequent modifier effects where the target loci are not necessarily functionally coherent as under the between-pathway model, and are identified by the BridGE algorithm as pathways that involve SNPs with an elevated number of SNP-SNP interactions. Specifically, BridGE tests each pathway-level interaction structure to assess enrichment for SNP-SNP interactions based on three statistics (χ2_global_, χ2_local_ and p_perm_ for BPM and WPM) (See Methods) [23]. BridGE also implements multiple disease models (based on the assumption that the alleles increasing susceptibility to the disease are recessive, dominant or additive) [23] and discovers interactions associated with both increased and decreased risk of the disease of interest.

### Analysis of genetic interactions in the BPC3 and CGEMS cohorts

We first applied our BridGE approach to the BPC3 and CGEMS cohorts (phs000812 and phs00147, respectively). These cohorts are both comprised of European Americans with genotypes measured using a common array platform (Illumina HumanHap550, Table 1), which provides a robust basis for replication analysis. We note that despite a common patient ethnic group, distinct disease populations are represented. The BPC3 cohort is comprised exclusively of women with ER-negative breast cancer while the CGEMS cohort consists of women with invasive, post-menopausal breast cancer. Previous studies suggest both unique and overlapping risk factors for ER negative and other breast cancers [47].

#### Discovery of between pathway interactions in BPC3 cohort (European)

Focusing first on identifying between-pathway model (BPM) interactions, we applied BridGE to the BPC3 cohort [38]. At a false discovery rate of 0.25, we identified 18 between-pathway interactions, corresponding to 11 distinct pathway pairs after removing redundancy (S1 Table). All 11 interactions were associated with increased risk and were discovered under a combined dominant/recessive model, which integrates SNP-SNP interactions arising from either a recessive or dominant disease model. Across the 11 discovered BPMs, there were 19 total pathways involved in these pathway-pathway interactions, and many of them were clearly relevant to the biology of breast cancer. For example, we found evidence for a genetic interaction between the steroid hormone biosynthesis pathway (Reactome) and a gene set associated with acute myeloid leukemia (Fig 2A). This gene set was obtained from KEGG and was created based on literature curation of the genetic events that are known to be crucial for leukemic transformation (many of them identified through somatic mutations observed for leukemia). The basis for our discovery of this interaction was an elevated density of SNP-SNP interactions bridging genes in these two pathways relative to the background density. Specifically, we observed a density of 0.05 of weakly significant SNP-SNP pairs relative to an expected background of 0.03 in the entire network, which was highly significant based on a null distribution estimated from 200,000 SNP label permutations (*p* = 1.0 × 10^−5^) (Fig 2B). This BPM was associated with increased risk, which means that for each SNP-SNP interaction pair supporting the BPM, individuals that were either homozygous (recessive and dominant models) or heterozygous (dominant only) for the minor allele at two loci of interest were enriched among the cases relative to the controls. None of the individual SNP-SNP interactions we identified were significant in individual pairwise tests (min. FDR = 0.94) (Fig 2C). Furthermore, although several of the individual SNPs supporting this BPM exhibited moderate univariate association (5.0 × 10^−4^ ≤ *p* ≤ 0.05) with breast cancer incidence (21/139 in AML, 4/38 in SHB), respectively, none of them would reach a standard level of genome-wide significance (*p* ≤ 10^−8^) suggesting that accounting for interaction between combinations of different common variants may contribute significantly to breast cancer heritability.

The steroid hormone biosynthesis pathway represented a major hub among our discoveries for the BPC3 cohort, appearing in 8 of the 11 discovered BPMs (Fig 3A). The steroid hormone biosynthesis pathway consists of a combination of cytochrome P450 heme-containing proteins and hydroxysteroid dehydrogenases that are responsible for converting cholesterol into active steroid hormones [44]. Previous studies have found an association between higher levels of endogenous estrogens, progesterone, cortisol, and androgens and higher incidence rates of breast cancer [48–50] [51]. Several individual genetic variants that modulate steroid hormone biosynthesis levels have been explored in relation to breast cancer risk, perhaps most notably a variant in the CYP11A1 enzyme [48]. Our results suggest that several common variants in this pathway may contribute to risk of breast cancer through genetic interactions with several other pathways.

#### Replication analysis of BPC3 interactions in CGEMS cohort (European)

We next examined whether significant between-pathway interactions discovered in the BPC3 cohort replicated in the CGEMS cohort (see Methods for details). BPMs were tested for replication in the independent cohort using our previously described approach [23]. The steroid hormone biosynthesis and the AML gene set (SHB-AML) BPM interaction described above was nominally significant (*p* ≤ 0.05) by all three measures of significance. Another two BPMs, both involving interaction of the steroid hormone biosynthesis pathway with either Na+/Cl- dependent neurotransmitter transporters or Amine compound SLC transporters, were also nominally significant by 2 of the 3 measures in the CGEMS cohort (S2A Table). In addition to evaluating the significance of the replication statistics for the individual BPMs, we also found that the overall degree of replication of the entire set of BPMs based on 10 random sample permutations was significant (S2B Table, fold-change >15, *p* = 0.0043).

We further investigated whether the individual SNP-SNP interactions supporting the discovered pathway-level interactions between the steroid hormone biosynthesis pathway and the AML gene set were similar across cohorts. The set of overlapping SNP-SNP interactions supporting this pathway-pathway interaction across the BPC3 and CGEMS cohorts was relatively small (6 SNP-SNP interactions in common, S3 Table). We observed more agreement between the cohorts when considering genes implicated by the interactions instead of SNPs (Fig 3B and 3C). Nonetheless, these results suggest that despite the common pathway-level interaction supported in both cohorts, distinct combinations of SNPs confer disease risk between these populations. This idea is consistent with the between pathway interaction model and may explain why success in discovering statistically significant SNP-SNP level interactions is limited in most standard GWAS cohorts.

#### Evidence for link between AML-associated genetic risk loci and breast cancer

Given the replication of the AML gene set and the steroid hormone biosynthesis pathway, we investigated the potential connection between AML genes and breast cancer risk. Interestingly, several previous studies have explored the link between breast cancer and AML and show that patients surviving breast cancer tend to exhibit higher incidence of AML [52, 53]. Although it remains unclear if this higher incidence is linked to genetic lesions induced by therapies used to treat breast cancer, the enrichment of SNP-SNP interactions connecting AML-linked genes to the hormone biosynthesis pathway observed in our analysis suggests that common genetic factors may contribute to increased susceptibility to both diseases. Interestingly, the transcription factor RUNX1 is included in the AML gene set and was a major driver of the BPM we discovered (Fig 3, S1 Table). There is an established link between the Runt family of transcription factors (RUNX1, RUNX2, RUNX3), which have been identified as key drivers of AML and other cancers [54], and breast cancer. Specifically, RUNX1 is highly expressed in luminal and basal cells in normal breast tissue, but its expression is reduced in many breast tumors, and lower expression of RUNX1 has been used to predict breast cancer metastasis [55]. RUNX2 has been shown to play an oncogenic role in breast cancer [54, 56, 57] and its expression has been associated with the triple-negative cancers and correlates with poorer patient survival [56]. Our data suggest that subtypes of breast cancer may share a common genetic basis given the fact that we observe an enrichment of SNP-SNP interactions connecting AML-linked genes to the hormone biosynthesis pathway associated with increased breast cancer risk.

Given the appropriate cohort with access to AML incidence post breast cancer, this hypothesis could be directly tested. We predict that there should be a subpopulation of patients whose breast cancer is due to interactions between AML-associated genes and variants in the hormone biosynthesis pathway. For these patients, we would expect an increased incidence of AML relative to other individuals with breast cancer regardless of whether they were treated with chemotherapy. Such a finding could have clinical utility because the relevant variant combinations could be used as a diagnostic marker to avoid administering chemotherapy to women who harbor a subtype of breast cancer that predisposes them to AML. Interestingly, we did identify a recent study that provides indirect support for this hypothesis [58]. This study focused on a set of women who developed leukemia after chemotherapy for breast cancer and identified germline risk factors enriched among these patients. The study concludes that these factors predispose those individuals to chemotherapy-induced leukemia. The authors also note previous reports of secondary AML diagnoses following only surgery or radiation treatment for breast cancer [59–62], supporting the idea that germline risk for AML within breast cancer patients even without exposure to chemotherapy may be a factor in the observed prevalence of AML among breast cancer survivors.

Although the number of common individual SNP-SNP interaction pairs supporting this pathway-level interaction was relatively small between these two cohorts, the set that does overlap provides a starting point for more in-depth analysis. The gene- and corresponding SNP-pairs that supported the discovery of this BPM in both cohorts include: RARA-HSD11B1, RAF1-HSD11B1, LEF1-HSD11B1, ZBTB16-HSD11B1, FLT3-STARD4, PIK3R3-CYP19A1; corresponding SNP-SNP pairs: rs4077125-rs742375, rs6442323-rs11119343, rs4956041-rs11119343, rs7118530-rs12143281, rs1933437-rs42670, rs3845301-rs3751586. Notably, a search of the NHGRI-EBI GWAS catalog [63] reveals that none of the genes or the SNPs involved in these pairs have been previously associated with breast cancer despite the clear relevance of the corresponding genes to cancer and evidence of genetic interactions in two independent cohorts. More investigation of the potential relevance of these interactions would be worthwhile.

While the steroid hormone biosynthesis-AML pathway interaction has the strongest support across these two cohorts, other pathways that we found to interact with the steroid hormone biosynthesis pathway were also relevant to breast cancer. For example, one of the other risk-associated BPMs that replicated in the CGEMS cohort connected the steroid hormone biosynthesis pathway and an amine compound SLC transporter gene set (Reactome). A recent study showed that amino acid transporters (e.g. SLC6A14) were upregulated in tumors of epithelial origin, including breast cancer, and suggested them as a possible new target for cancer treatment [64]. Another interaction connected the steroid hormone biosynthesis pathway to a Type II diabetes gene set (KEGG), which is interesting given previous findings that women with type II diabetes have elevated risk of breast cancer. This interaction suggests a potential genetic basis for this comorbidity [65].

#### Discovery of within pathway interactions and pathway interaction hubs in BPC3 and CGEMS

In addition to between-pathway interactions, the BridGE approach can also be used to identify single pathways that are enriched for SNP-SNP interactions mapping to multiple genes within the same pathway (within-pathway model, WPM) or pathways that are enriched for SNP-SNP interactions across the entire genome (pathway hub model, PATH). Applying BridGE to the BPC3 and CGEMS cohorts, we identified 2 WPM and 3 PATH interactions from the BPC3 cohort and 2 WPM and 4 PATH interactions on the CGEMS cohorts (FDR≤0.25, S1 Table, S4 Table). For example, in the CGEMS cohort, we found that the PKA activation pathway (Reactome) was enriched for risk-associated WPM and PATH interactions. Both types of interactions were replicated in a second cohort with permutation p-values of 0.004 and 0.015 respectively (MCS Japanese, see JPN517 in S5 Table). Previously, PKA activation has been associated with prognosis and resistance to certain therapies against breast cancer (e.g. [66], [67]). It is worth noting that the validation of this PKA activation was based on a cohort consisting of Japanese women while our discovery was completed on women of European ancestry. This suggests that the PKA activation pathway may be a common breast cancer risk factor across different populations.

### Application of BridGE method to four additional non-European breast cancer cohorts

In addition to detailed analysis of the two European cohorts described above, we also applied BridGE to four additional cohorts, for a total of six cohorts: MCS (JPN, LTN, AA) and SBCGS (CHN) (Table 1). The JPN cohort was genotyped using the Illumina Human 1M platform, and thus, to facilitate comparison between the JPN and CHN cohorts, we used the imputed SNPs in the CHN cohort (Affymetrix 6.0 platform) to ensure enough common SNPs across two cohorts for BridGE analysis. For the CHN cohort, we attempted discovery both from the original genotypes as well as the imputed profiles. Results on the JPN cohort were originally reported in our companion paper [23], but are analyzed in the context of the other cohorts discussed here. BridGE was applied to discover between-pathway (BPM), within-pathway (WPM), and pathway hub (PATH) interactions independently from three out of four additional cohorts (Table 2, MCS AA cohort is omitted as it did not yield significant discoveries).

**Table 2:** Summary of discoveries across five breast cancer cohorts.

Study	Race	Disease Model	Interaction Type	min(fdr)	Number of significant discoveries (non-redundant)				
					fdr≤0.05	fdr≤0.1	fdr≤0.15	fdr≤0.2	fdr≤0.25
BPC3	EUR	Combined	BPM	0.25					18 (11)
			WPM	0	1 (1)	2 (1)	2 (1)	3 (2)	3 (2)
			PATH	0	5 (2)	8 (2)	10 (2)	10 (2)	13 (3)
CGEMS	EUR	Dominant	BPM	0.35					
			WPM	0.23					3 (2)
			PATH	0.13			5 (4)	5 (4)	5 (4)
MCS	JPN	Dominant	BPM	0.15			86 (37)	108 (43)	124 (48)
			WPM	0.4					
			PATH	0.1		2 (2)	2 (2)	2 (2)	2 (2)
MCS	LTN	Dominant	BPM	0.31					
			WPM	0.92					
			PATH	0.1		1 (1)	1 (1)	1 (1)	1 (1)
SBCGS	CHN	Combined	BPM	0	12 (7)	28 (19)	38 (21)	73 (34)	84 (37)
			WPM	0	2 (1)	2 (1)	2 (1)	3 (1)	3 (1)
			PATH	0.1		1 (1)	2 (1)	2 (1)	2 (1)

Indeed, we were able to find genetic interactions in three of the four additional cohorts, although the number of interactions identified varied across cohorts as did the corresponding model (BPM, WPM, or PATH) (S6 Table, S7 Table and S8 Table). Notably, the SBCGS CHN cohort, the largest of all cohorts we analyzed, produced a large number of discoveries (S7 Table). For example, at an FDR of 0.25, we discovered 37 distinct BPMs, 1 WPM, and 1 PATH interaction. Several of these involved DNA repair pathways. In particular, the base excision repair pathway (Reactome) was involved in 6 of the 39 genetic interactions we discovered and included interactions with other pathways such as an adipocytokine signaling pathway (KEGG), ubiquitin-mediated proteolysis (KEGG), and a renal cell carcinoma gene set (KEGG) (S7 Table). Because some of the most well-known risk factors for breast cancer, e.g. BRCA1/BRCA2, PALB2, and ATM, are involved in DNA repair [37, 68], the prominence of this pathway is not surprising. Our finding suggests that these pathways are frequent modifiers in this population.

### Consensus analysis of pathway-level genetic interactions across five cohorts

**Fig 4:**
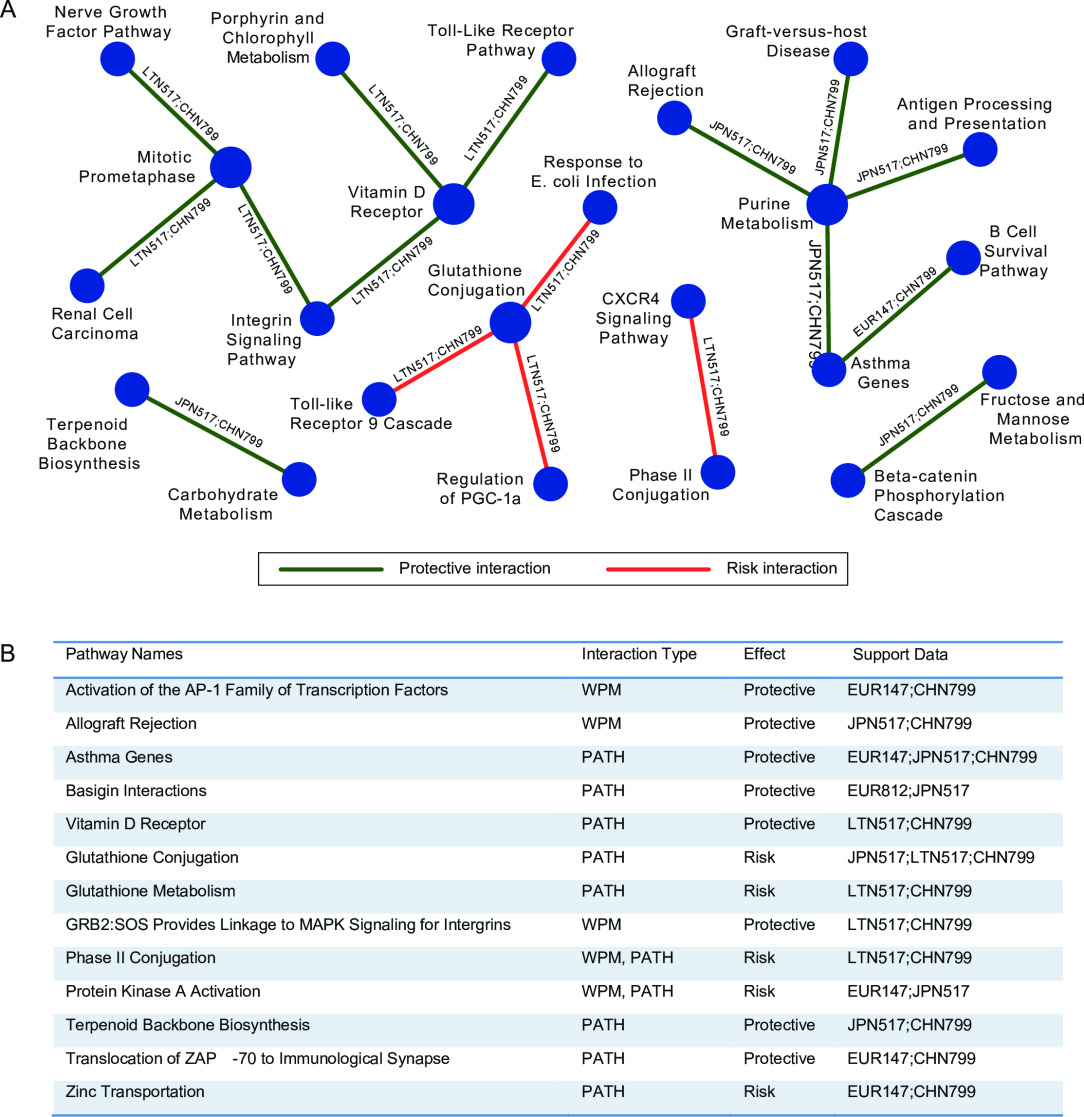
Consensus summary of pathway-level interactions discovered from the 6 GWAS breast cancer cohorts. (A) Network view of the most significant between-pathway interactions (BPM) (geometric mean p ≤ 5.0 × 10^−5^) that are supported by at least two cohorts. The supporting cohorts are indicated by the edge labels. (B) List of all within-pathway interactions (WPM) and hub pathways (PATH) that are most significant (geometric mean p ≤ 5.0 × 10^−3^) and supported by at least two cohorts.

Most of the significant genetic interactions discovered across the five cohorts were unique to each cohort, suggesting that the strongest genetic interactions are distinct in each population and may reflect the broad set of ethnicities represented by these cohorts. However, we reasoned that there may also be common genetic interactions underlying breast cancer risk across diverse populations, and that if we performed joint discovery across these diverse cohorts, we may be able to detect such universal risk factors. We anticipated that pathway-level interactions with moderate significance in individual cohorts that were consistently identified across multiple populations would be highly significant when analyzed together. Applying this principle, we extended our BridGE approach to enable joint analysis of between-, within-, and hub-pathway interactions across multiple cohorts. Significance of BPMs, WPMs, or PATH interactions with support across multiple datasets was assessed through resampling of the pathway-level statistics from 10 sample permutations (case-control label permutations) we ran for all five cohorts (see Methods for details). Indeed, this analysis identified a set of BPM, WPM and PATH genetic interactions with significant support across multiple different cohorts (Fig 4, S5A, S5B, S5C and S5D Table). For example, for BPM interactions, at a stringent joint significance threshold (*p* ≤ 1 × 10^−5^, see Methods for details), we identified 17 BPMs with support in multiple cohorts, which was significantly more than random expectation based on a permutation-derived null distribution (*p* = 0.02, S5D Table, see Methods for details). Similar analysis for WPM and PATH interactions suggested greater than expected coherence across the cohorts as well (S5D Table).

**Fig 5:**
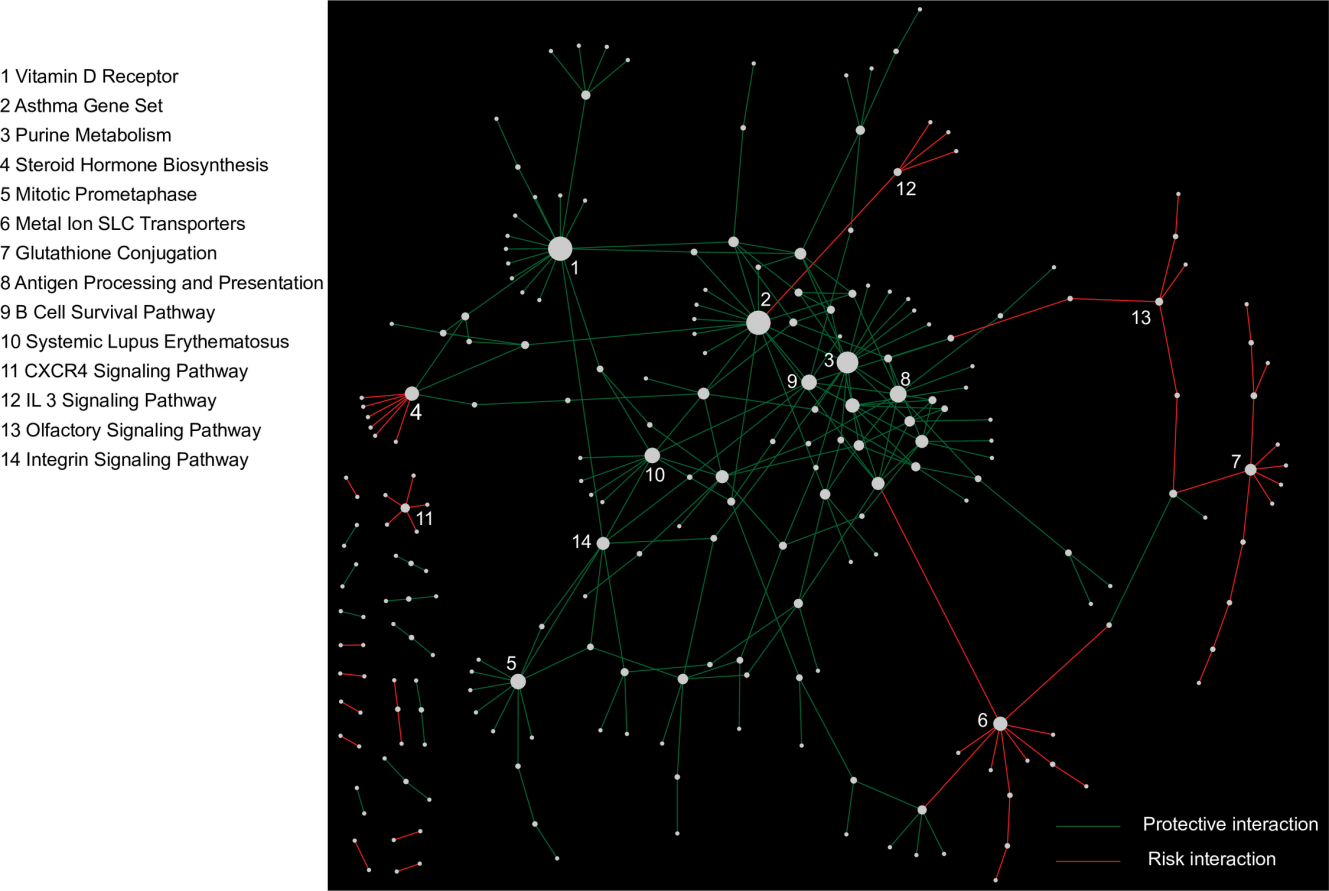
Network view of the between-pathway interactions (BPM) from the consensus analysis. All BPMs satisfying a geometric mean p ≤ 5.0 × 10^−4^ threshold from consensus analysis are plotted. Red edges indicate interactions associated with increased breast cancer risk while green edges indicate interactions associated with decreased risk. Node size is proportional to the number of BPMs connected to each pathway. Several of the highly connected pathways are labeled by numbers, and their corresponding pathway names are listed. The complete information for these pathways can be found in S5 Table.

We visualized the complete set of BPM interactions as a network to explore the relationship between the discovered interactions (Fig 5). Several interesting breast cancer-relevant pathways emerged as part of this analysis, including a vitamin D receptor pathway that appeared to act as a consensus interaction hub by connecting several significant BPMs with support from at least two cohorts each. This hub’s existence suggests that the vitamin D receptor pathway is an important modifier of breast cancer risk. All of these interactions with the vitamin D receptor pathway were associated with protective effects (decreased risk of disease) and included interactions with integrin signaling and the toll-like receptor signaling pathway (Fig 4A). Vitamin D is a secosteroid hormone, and several previous studies have explored the potential protective effect of vitamin D levels on breast and other cancers [69, 70]. Interestingly, despite substantial interest, studies on the protective effects of vitamin D in cancer have produced mixed results [69]. Our observation that the vitamin D receptor pathway participates in many genetic interactions may suggest that only specific subsets of patients will benefit from increased dosage of vitamin D, which is consistent with these findings. These interactions were primarily supported in the MCS LTN and SBCGS CHN cohorts. Another pathway, the glutathione conjugation pathway (Reactome) (Fig 4 and Fig 5), emerged as the single strongest consensus pathway interaction hub (PATH) associated with increased breast cancer risk, with support in three of the five cohorts examined (MCS LTN, MCS JPN, and SBCGS CHN). Several between-pathway interactions were associated with the glutathione conjugation pathway in the consensus analysis as well. With additional cohorts, these discoveries could be assessed for replication beyond our consensus analysis, which would further increase confidence.

### Glutathione conjugation as a common breast cancer modifier

Glutathione S-Transferases (GSTs) comprise a large and conserved family of enzymes that catalyze conjugation of reduced glutathione (GSH) to a variety of substrates [71]. GST-mediated conjugation of glutathione often leads to formation of less reactive products and, as a result, GSTs play an important protective role in the detoxification of toxins and reactive oxygen species produced as a result of oxidative stress [71]. Non-enzymatic roles have also been reported, whereby GSTs modulate specific cell functions through physical interaction with specific proteins and lipids in a GSH independent manner [71].

**Fig 6:**
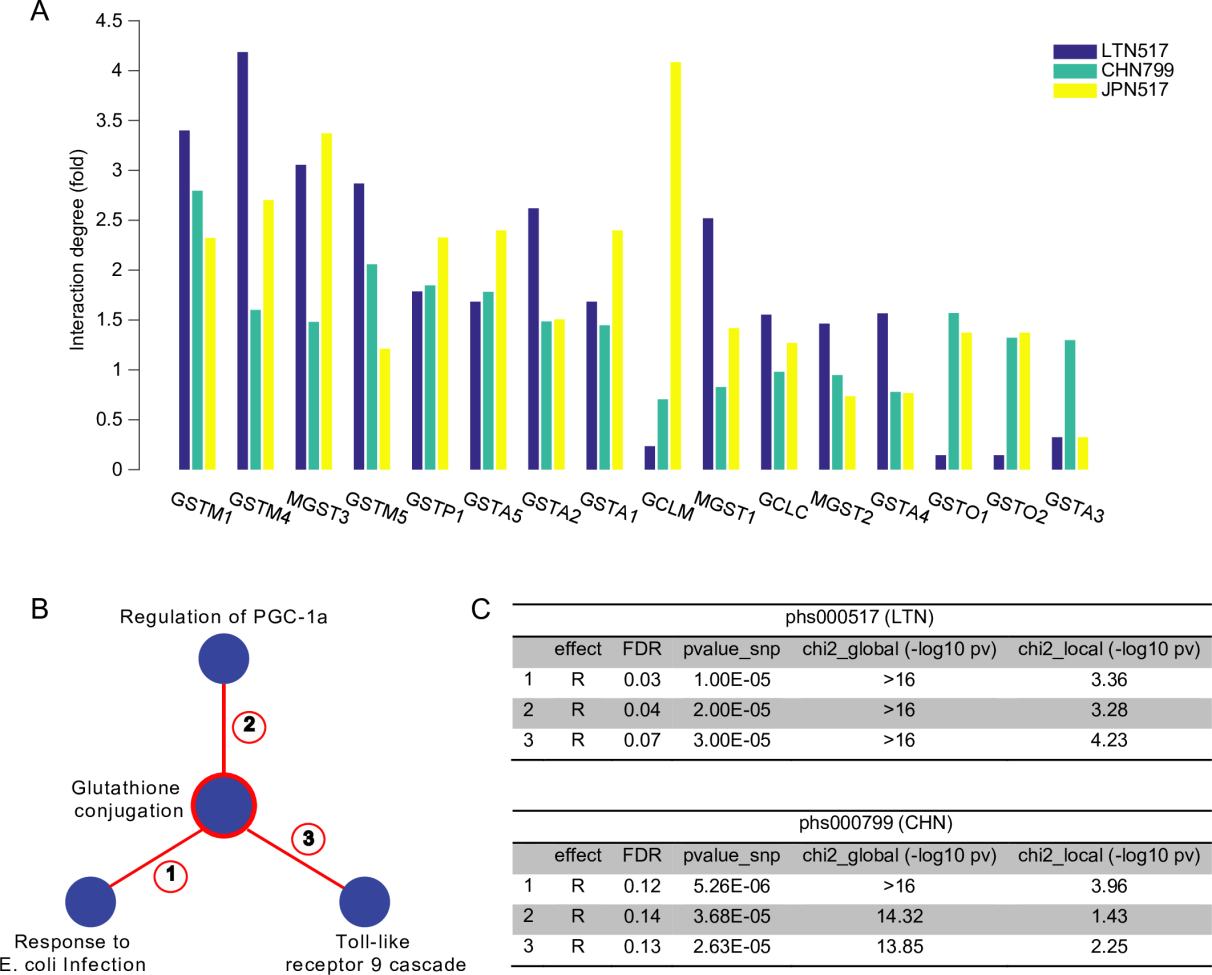
Consensus interactions with the glutathione conjugation pathway. (A) Gene interaction degree (fold enrichment) of all glutathione conjugation genes in the three cohorts that support a PATH interaction for the glutathione conjugation pathway (LAT517, CHN799 and JPN517). (B) Between pathway interactions associated with glutathione conjugation that are significant (*FDR* ≤ 0.25) in both LAT517 and CHN799 datasets. The red edges indicate they are all associated with increased risk of breast cancer. (C) Detailed statistics for the between pathway interactions shown in (B).

Based on their broad enzymatic and non-enzymatic functions, GSTs have been identified as important targets for anti-inflammatory and anti-tumor drug therapies [71]. Indeed, several GST isoenzymes have been associated with various forms of cancer. For example, the GSTM family of isoforms has been the focus of more than 500 studies examining associations between GSTM genotypes and various malignancies. One of these studies suggested that homozygous deletion of GSTM1 is associated with protective effects against breast cancer [72] while other studies proposed that GSTM1 null alleles have a modest effect on lung cancer [73]. Polymorphisms in another GST isoenzyme, GSTP1, have also been shown to modify response to chemotherapy in patients with colorectal cancer and multiple myeloma [74, 75], and GSTP1 was shown to influence risk of acute myeloid leukemia in patients successfully treated for breast cancer, non-Hodgkins lymphoma, Hodgkins and ovarian cancer [76]. Furthermore, human tumor cell lines can overexpress GSTP1, GSTA and GSTM isoenzymes [77]. In fact, GSTP1 overexpression is considered a major cancer biomarker that can influence both disease development and treatment [77]. For example, GST overexpression can lead to enhanced GSH conjugation and inactivation of chemotherapeutic agents [71] as well as aberrant regulation of cell growth and apoptosis signaling pathways caused by direct binding and sequestration different protein and hormone ligands [71, 77–82]. Indeed, our systematic analysis to identify between-pathway interactions involving the glutathione conjugation pathway revealed a clear relationship between GSTs and cancer-related signaling pathways (Fig 6B and 6C, S10 Table).

Given the discovery of the glutathione conjugation pathway as a pathway interaction hub (PATH) from our consensus analysis, we performed a full analysis of the three relevant cohorts to focus on discovering significant between-pathway interactions that specifically involved the glutathione conjugation pathway. By focusing on just this pathway, we further reduced the number of hypothesis tests to improve our power to discover specific pathways interacting with glutathione conjugation. This approach was successful for two of the three cohorts (MCS LTN, SBCGS CHN) and produced 17 and 77 interactions at *FDR* ≤ 0.25 (S10 Table), respectively.

Strikingly, 3 of these BPMs were independently discovered in both cohorts: regulation of PGC-1a, toll-like receptor 9 cascade and response to E. coli infection (Fig 6B and 6C). One of these pathways, PGC1A (also called PPARGC1A) regulates the activity of numerous transcription factors that regulate cell growth and proliferation. These transcription factors include PPARγ (Peroxisomal Proliferator-Activated Receptor γ), PPARa (Peroxisome proliferator-activated receptor alpha), GR (glucocorticoid receptor), THR (thyroid hormone receptor) and estrogen receptors (Biocarta, [83]). Unsurprisingly, variants in PPARGC1A, PPARGC1B, PPARγ and PGC1a have been associated with familial as well as alcohol-related breast cancer risk [84, 85]. In addition, PPARγ is upregulated in colon and breast cancer cells [86, 87] and relationships between PGC1a expression levels in breast tumors and clinical outcome have also been reported [88, 89].

Importantly, a mechanistic link between the PGC1a pathway and the glutathione conjugation pathway is well established, supporting the interactions we discovered and suggesting increased breast cancer risk in patients carrying variants in both of these pathways [71]. Specifically, PPARγ is activated by binding to its ligand 15-deoxy-Δ-prostaglandin J_2_ (15d-PGJ_2_), a potent cyclopentanone [78]. 15d-PGJ_2_ biosynthesis requires GST [71] and, in addition to its production, GST also regulates 15d-PGJ_2_ activity by directly binding to both GSH-conjugated and unconjugated forms of 15d-PGJ_2_ and sequestering the ligand in the cytosol away from its nuclear target, PPARγ [78, 81]. Indeed, stable expression of GST in a breast cancer cell line inhibited PPARγ-dependent gene expression [78]. Other studies have also shown that GST can modulate the activity of various signaling and metabolic pathways in a similar manner suggesting that sequestration by GST may represent a general mechanism for regulating pathway function [71, 77, 79, 80, 82]. Such a regulatory role is consistent with our discovery of the glutathione conjugation pathway as a pathway interaction hub (PATH) (Fig 5A) and its interactions with a substantial number of pathways known to control cell growth and proliferation.

Another interaction involving glutathione conjugation included the Toll-like receptor 9 (TLR9) pathway (Fig 6B). TLR9 is known to control the innate immune response by detecting foreign DNA from microbial or other sources [90], And has been extensively studied in the context of breast cancer [91]. TLR9 expression has been measured in normal epithelial cells of the mammary gland as well as epithelial cancer cells and fibroblast-like tumor cells [91]. TLR9 has also been shown to have prognostic significance, specifically in triple negative breast cancers where low TLR9 expression is associated with shorter disease-free-specific survival. The link between TLR9 and glutathione conjugation is unclear, but the established relevance of both pathways to breast cancer suggests that this interaction is worth further study. In general, the independent discovery of several genetic interactions involving the glutathione conjugation pathway across multiple cohorts suggests that it likely acts as a common modifier for other risk factors.

### Summary and proposed future work

We described the application of our recently developed method, BridGE, to several breast cancer cohorts. We found significant discoveries across 5 of the breast cancer cohorts examined, suggesting that genetic interactions indeed play a role in determining breast cancer risk. Our approach leverages the key observation from reverse genetic screens in yeast, which observed that genetic interactions often form dense clusters in which they bridge across two pathways, or connect pairs of genes within the same pathway. This observation about local structure prevalent in the yeast genetic interaction network provides a powerful basis for discovering interactions in human populations. Our results here demonstrate that this approach can shed new light on risk factors for breast cancer.

We note that many of the pathways involved in genetic interactions reported here are novel and have never been implicated as genetic risk factors for cancer. For example, if we consider only BPM, WPM or PATH interactions passing a conservative cutoff of FDR < 0.05, we discovered a total of 25 pathways across the five cohorts (S11A Table). Based on the dbGaP GWAS catalog, traditional univariate analyses have identified 172 distinct SNP variants associated with breast cancer (p ≤ 1.0 × 10^−5^) from published GWAS. Mapping these variants to nearby genes and then to pathways reveals that only 9 of the 25 pathways involved in the genetic interactions reported here include a gene for which a SNP had previously been reported, suggesting that the remaining 16 of 25 have not been previously implicated through germline genetic analysis. Thus, despite their clear relevance to breast cancer, the majority of genetic interactions reported here represent novel mechanisms underlying genetic risk of breast cancer relative to previous studies of single variants. Interestingly, the BridGE-discovered pathways also cover many of the previously reported SNPs. Of the 172 unique GWAS SNPs mentioned above, 47 can be mapped to our collection of 833 pathways and 34 of these 47 (72%) map to at least one of the set of BridGE-discovered pathways (FDR ≤ 0.25), suggesting that most pathways linked to previously identified SNPs from single locus analysis are also involved in genetic interactions.

There were a large number of pathway-level interactions unique to individual cohorts we examined, suggesting that interactions can be contributed by a broad range of mechanisms and likely vary substantially across different human populations. We did, however, find evidence for a core set of interactions with support across multiple populations. Specifically, significant interactions involving glutathione conjugation, vitamin D receptor, purine metabolism, mitotic prometaphase, and steroid hormone biosynthesis pathways were discovered across different cohorts, suggesting these pathways may act as important general modifiers of breast cancer.

There are several other interesting directions for future work based on the results presented here. First, one of the main inputs of the BridGE method is the definition of pathways, for which it then discovers genetic interactions. Of course, the quality and utility of the genetic interactions discovered depend on the quality of the input pathway definitions. We expect that there are several pathways highly relevant to breast cancer that are not yet well-understood or at least not well-captured by current pathway databases. As these pathway definitions improve, the BridGE approach will improve in terms of its power in discovering interactions. In the context of breast cancer, there is a wealth of functional genomic data (e.g. gene expression profiles) that could directly inform the definition and further refinement of pathways. Leveraging these unbiased data to improve the input pathways before running BridGE would be worthwhile.

Another limitation of the BridGE approach is the resolution of the discovered interactions. The genetic interactions reported in this study were all discovered at the pathway level (i.e. between or within-pathways). The premise of the method, and indeed the reason we are even able to discover genetic interactions, is that while power to detect individual pairs of SNPs with disease association is low, these associations can be discovered at the pathway level. Because of this, it is typically difficult to pinpoint individual SNPs or combinations of SNPs for further investigation. For example, for the steroid hormone biosynthesis-AML gene set interaction, the BPM was discovered on the BPC3 cohor and replicated on the CGEMS cohort. However, the overlap in the individual SNP-SNP interactions supporting these BPMs in the different cohorts was relatively small. This likely reflects both the fact that our power for detecting the actual SNP-SNP interactions underlying the association is limited as well as the fact that the actual SNPs contributing interactions between these pathways can be highly heterogeneous. Our analysis of the glutathione conjugation pathway discovery provides some hints at how to approach this challenge. Once BridGE identified the glutathione conjugation pathway as a risk factor in several cohorts, we computed the density of SNP-SNP interactions connecting each gene in the pathway. This did highlight substantial differences in the SNP-SNP interaction density contributed by each gene, providing some clues as to which individual SNPs have the strongest contributions to the pathway-level trend (Fig 6A). Consensus analysis of consistent SNP-level interactions across independent cohorts, much like we performed at the pathway level, could also be an effective strategy for narrowing the focus to individual variant combinations. In general, improved methods for further dissecting pathway-level genetic interactions to identify individual SNPs or pairs of SNPs responsible for a pathway-level interaction would be of interest.

Finally, another direction worth further investigation is analysis of the clinical relevance of the discovered interactions. We expect that, at least in some cases, the genetic interactions predisposing individuals to breast cancer will influence the prevention, progression, and optimal treatment of the disease. Application of our method to large cohorts with the corresponding clinical information and development of predictive modeling approaches that leverage both pathway-level and SNP-level information from the discovered genetic interactions to model clinical features will be a focus of future work.

## Methods

### Datasets used

#### U4C designated breast cancer GWAS datasets

The National Cancer Institute provided seven breast cancer GWAS datasets for the U4C Stimulating Innovation in Breast Cancer Genetic Epidemiology Challenge: phs000812.v1.p1, phs000147.v3.p1, phs000517.v3.p1, phs000799.v1.p1, phs000851.v1.p1, phs000912.v1.p1, phs000383.v1.p1. Among them, we focused our analysis on four datasets: phs000147.v3.p1, phs000812.v1.p1, phs000517.v3.p1, and phs000799.v1.p1. A brief summary of these four datasets can be found in Table 1.

#### Other datasets

We used Hapmap Phase III data [92] as our population reference data to filter out sample outliers. We used pathways from the MSigDB v3.0 C2 curated collection [93] as our candidate pathways. We required each pathway to have at least 10 genes and at most 300 genes, and at least 10 SNPs and at most 300 SNPs after mapping the pathways to SNP level. A power analysis with respect to the pathway size suggested that our power would be limited for pathways with fewer than 10 genes [23]. Gene sets with too many genes are unlikely to provide actionable information as they most likely do not represent specific biological functions. Thus, including these pathways in our testing set only exacerbates the multiple testing issues without a strong likelihood that we can actually discover an interaction for them. Among the 833 pathways from our pathway collection, only 3 had less than 10 genes and 9 had more than 300 genes. A SNP was mapped to all genes that overlap with a +/- 50kb window centered at the SNP, and then mapped to pathways to which the corresponding gene(s) were annotated.

### A brief overview of the BridGE method

The details of the BridGE method are described in our separate paper [23], but we provide a brief overview of the approach here. Because there is not enough power to detect individual SNP-SNP interactions from most GWAS studies, based on the observation from yeast reverse genetic screen that genetic interactions often form dense clusters bridging across two pathways, or connect pairs of genes within the same pathway, we developed a method to specially search for pathway-level interactions. More specifically, BridGE searches for three different structures:

Between-pathway model (BPM): Between-pathway interactions occur when two pathways impinge on a common function required to maintain a healthy (non-disease) state; because the two pathways can functionally compensate for each other, the disease phenotype only occurs when genetic perturbations occur in both pathways in the same individual.

Within-pathway model (WPM): Under the within-pathway model, a single genetic variant partially disables a pathway’s function and additional partial loss of function variants affecting the same pathway result in a complete loss of pathway function, leading to a disease state.

Hub pathway model (PATH): Pathway hubs correspond to pathways with frequent modifier effects where the target loci are not necessarily functionally coherent as under the between-pathway model.

Briefly, the BridGE approach involves the following five main components [23]:

(1) Data processing consisting of sample quality control, adjustment for population structure between the cases and controls to avoid false discoveries due to population stratification, and control for linkage disequilibrium (LD) by pruning the full set of SNPs into an unlinked subset, as LD could otherwise result in spurious BPM or WPM substructures. (2) Construction of SNP-SNP interaction networks based on SNP pair-level genetic interactions scored under different disease model assumptions (additive, recessive, dominant or combinations of recessive and dominant models). (3) A low-confidence, high-coverage interaction network is derived by applying a lenient threshold to the SNP-SNP interaction network. (4) Pairs of pathways from predefined gene sets are tested for BPM or WPM enrichment of SNP-SNP pair interactions with a chi-squared test. The observed density is evaluated for significance based on comparisons to the global density (χ2_global_), the marginal interaction density of the two pathways (χ2_local_), as well as a permutation test (p_perm_) conducted by randomly shuffling the SNP-pathway assignment (e.g. 100K~200K times). (5) Pathway-level statistics are assessed for significance after correction for multiple hypothesis testing. Each pathway-level interaction can be associated with either increased risk of disease (risk interaction: pairs of minor alleles linking two pathways are more frequent in the diseased population) or decreased risk of disease (protective interaction: pairs of minor alleles linking two pathways are more frequent in the control population). So, for example, for the between-pathway model (BPM) the number of hypothesis tests evaluated by BridGE is two times the number of all pair-wise pathway-pathway interactions. A sample permutation strategy (e.g. permutation of the case-control labels 10 times) is used to estimate the false discovery rate accounting for multiple hypotheses testing. Further details of our methods are described in [23].

### Data processing for the U4C datasets

For each dataset analyzed, we followed these steps to perform quality control: (1) we used a standard PLINK (Purcell, et al. 2007) procedure to remove individuals with more than 5% missing values, and remove SNPs with more than 5% missing values, less than 5% minor allele frequency, or failed Hardy-Weinberg equilibrium test at 1.0E-6; (2) we checked relatedness among individuals, and for any pair of individuals that had a proportion IBD score greater than 0.2, one of them was removed from the study; (3) we removed subjects that were identified as population outliers based on multidimensional scaling (MDS) analysis after combining the study data with HapMap phase III data [92]; (4) we ensured balanced population structure between the cases and controls by matching each case with a control (implemented in PLINK with "—cluster—cc–mc 2"). All datasets analyzed were processed with these steps. Additional steps unique to each cohort are included in the sections that follow.

#### phs000812 Breast and Prostate Cancer Cohort Consortium (BPC3) and phs000147 Cancer Genetic Markers of Susceptibility Study (CGEMS)

For the two breast cancer European cohorts, BPC3 (phs000812) and CGEMS (phs000147), since there is a partial overlap in cases and controls between these two cohorts, we first applied identity by descent (IBD) analysis to recognize these overlapping individuals and removed them from the BPC3 (phs000812) cohort, while keeping them in the CGEMS cohort (phs000147).

#### phs000517 Multiethnic Cohort Study (MCS)

The phs000517 dataset has three population groups: JPN, LTN and AA. We used MDS analysis as described above to split this cohort into three sub-cohorts, one for each ethnic group. Samples that did not cluster within each group based on MDS analysis were filtered from the cohort. These three sub-cohorts are also labeled as JPN517, LTN517 and AA517 respectively.

#### phs000799 Shanghai Breast Cancer Genetics Study (SBCGS)

To allow for direct comparisons with results from the phs000517 cohort, we used imputed SNPs from the phs000799 cohort, which was genotyped using the Affymetrix 6.0 platform, to match the SNPs measured on the Human660W-Quad_v1_A and Illumina Human 1M platforms, which were used to genotype the phs000517 cohort. We ran our BridGE approach on both the non-imputed dataset and imputed dataset and reported discoveries for both. These imputed and non-imputed versions of the phs000799 dataset are referred to as “CHN799 imputed” and “CHN799 non-imputed”.

### Application of BridGE to discover significant BPM, WPM and PATH interactions in breast cancer GWAS cohorts

We applied the BridGE method to six different cohorts derived from four GWAS breast cancer studies (BPC3 phs000812, CGEMS phs000147, MCS phs000517, and SBCGS phs000799). Specifically, we tested pathway-level interactions (BPM, WPM and PATH) for 6 different cohorts (EUR812, EUR147, JPN517, LTN517, AA517, and CHN799), and for the CHN799 cohort, we used the both imputed and non-imputed data, independently. Details of the procedure used are described in the sections that follow.

For each dataset, we first ran pilot runs to find a proper set of parameters to be used for a full BridGE run. Specifically, we tested the four disease models (additive, recessive, dominant, or combinations of recessive and dominant models) with different network thresholds by performing a small number of SNP permutations (10,000) (SNP-pathway assignment was randomly permuted), and estimated which combination of disease model and network density cutoff was the most sensitive for each dataset. Based on the pilot results, a recessive/dominant combined disease model was chosen for BPC3 (network density = 0.06), SBCGS (network density = 0.04) for both imputed and non-imputed version; a dominant model was chosen for CGEMS (network density = 0.04), MCS JPN cohort (network density = 0.04), and MCS LTN cohort (network density = 0.02). For the MCS AA cohort, the pilot run suggested that we were unlikely to discover pathway-level interactions, so we did not apply a full BridGE run on this dataset in order to focus our computational resources on analysis of other cohorts. For all BridGE runs, we used supercomputing resources provided by the Minnesota Supercomputing Institute.

As described in [23], for the discovery of between-/within-pathway (BPM/WPM) interactions, three metrics are used to measure the significance of the density of SNP-SNP interactions: χ2_global_ and χ2_local_ are chi-square tests to measure whether the observed SNP-SNP interaction density between two pathways, or within a pathway, is significantly higher than expected globally (the overall network density), and locally (the marginal density of SNP-SNP interactions for any SNPs linked to genes in either of the two pathways). Additionally, a permutation test in which SNP labels are randomly reassigned is used to derive a third measure of significance (p_perm_). These permutations are used to establish a null distribution for χ2_global_ and χ2_local_ for each between-/within- pathway interaction. Finally, a false discovery rate is estimated for the entire set of between- or within-pathway interactions based on sample permutations in which the entire process is repeated under permutations of the case-control labels (χ2_global_, χ2_local_ and p_perm_) [23].

For the hub pathway interactions (PATH), a one-tailed rank-sum test was used to test if the SNPs linked to each pathway show significantly more interactions than non-pathway SNPs, in terms of interaction degree. The sample permutation and SNP label permutation procedure is same as the between- and within-pathway interaction discovery.

We reported all significant BPM, WPM or PATH interactions with FDR≤0.25. The summary table (Table 2) shows that all five of the datasets have significant BPM, WPM or PATH level interactions. Since many of the pathways overlap with each other, the total number of discoveries can be inflated by the fact that many overlapping pathway-pathway interactions reflect the several overlapping pathways. Thus, we also report the number of discoveries after filtering for redundancy among the pathway interactions [23], and the information on overlap is included in our supplemental files. Detailed discovery information for each cohort can be found in (S1 Table, S4 Table, S6 Table, S7 Table, S8 Table and S9 Table). All technical details of the BridGE method are described in [23].

### Replication analysis of discoveries in BPC3 and CGEMS

For significant pathway-level interactions identified from any of the two European cohorts (BPC3, CGEMS), we performed replication analysis. The disease models used for full interaction discovery in the BPC3 and CGEMS were different based on trends observed in the pilot runs (see details above), so for the replication analysis, we used the disease model with the discovery cohort and ran BridGE with 1000 SNP label permutations for all candidate pathway-level interaction. Ten sample permutations were also run for the replication cohort, just as in the discovery cohort.

Significant discoveries were tested for validation with two different approaches. The first approach checked for replication of the individual pathway-level interactions. For each pathway-level interaction (e.g. BPM, WPM, or PATH), we measured all three significance scores (χ2_global_, χ2_local_ and p_perm_) on the replication cohort and tested whether they met a nominal significance criteria (p ≤ 0.05). Of the discoveries from the BPC3 cohort, one of the 18 significant pathway-pathway interactions, the BPM connecting the steroid hormone biosynthesis pathway to an AML gene set, was significant (*p* ≤ 0.05) by all three measures. Two additional BPMs were significant (*p* ≤ 0.05) by two of the three significance measures (amine compound SLC transporter gene set and Na(+)- and Cl(-)-dependent neurotransmitter transporters) when tested for replication in the CGEMS cohort (S2 Table).

In addition to testing for replication of individual pathway-level interactions, we further investigated if the total number of replicating interactions among the entire set of discoveries was higher than expected by chance. This “set-level” replication analysis was done by resampling of the same amount of pathway-level statistics from all pairwise pathway interactions. Further details on replication procedures are described in [23].

### Comparison of pathways discovered by genetic interaction analysis with previously reported breast cancer risk loci

Based on the NHGRI-EBI GWAS catalog[63], there are 172 SNP variants (mapped to 134 genes) reported with strong association (p≤1.0 x 10^−5^) with breast cancer susceptibility. To measure the extent to which our approach produced new pathway-level insights about breast cancer susceptibility, we evaluated how many pathways in our collection were implicated basted on these 172 risk loci, and how many pathways discovered by BridGE analysis were novel relative to this set derived from traditional GWAS single variant analysis.

Of 172 SNPs linked to known breast cancer risk loci, 47 of these SNPs could be mapped to our collection of 833 pathways. Then we collected all pathways that were identified by BridGE in any of the breast cancer cohorts analyzed here with a conservative FDR cutoff (*FDR* ≤ 0.05), which yielded a total of 25 unique pathways either from significant BPM, WPM or PATH discoveries. Among these pathways, 9 were in common with the pathways already implicated by at least one known breast cancer risk locus. Thus, our analysis of genetic interactions by BridGE has implicated 16 new pathways (FDR ≤ 0.05) as playing a role in breast cancer susceptibility. We listed all unique pathways resulted from the less stringent FDR cutoff (FDR ≤ 0.25) in S11A Table.

### Consensus analysis to evaluate pathway-level interactions across multiple cohorts

Although we found that many pathway-level interactions discovered by BridGE were relevant to breast cancer, the most significant pathway-level interactions discovered from each cohort were relatively unique, suggesting that the strongest genetic interactions in each population are distinct. However, we observed that interactions discovered in one cohort often exhibited strong signals in additional cohorts even though they did not meet the stringent threshold required for discovery significance in a single cohort. Thus, we developed a modified version of BridGE to enable joint discovery of pathway-level interactions across cohorts to enable the discovery of these moderately significant, but consistent interactions.

More specifically, we first ran the standard version of BridGE on 4 different GWAS datasets (6 total cohorts), and we summarized each pathway-level interaction based on its permutation p-values (p_perm_) across all cohorts. We then selected pathway-level interactions that were nominally supported by at least two cohorts, for which we required that all test scores (χ2_global_, χ2_local_ and p_perm_ for BPMs and WPMs, degree rank-sum test and p_perm_ for PATH) be nominally significant (*p* ≤ 0.05). For each surviving pathway-level interaction, we computed the geometric mean of the p-values of all individual cohorts that met the nominal significance requirement. These criteria produced a total of 3930 consensus between-pathway interactions (BPM), 76 within-pathway interactions (WPM), and 59 hub pathway interactions (PATH), which were sorted based on the aggregate p-value (see S5 Table). The most significant between-pathway interactions (geometric mean ≤ 5.0 × 10^−5^), within-pathway interactions (geometric mean *p* ≤ 5.0 × 10^−3^), and hub pathway interactions (geometric mean *p* ≤ 5.0 × 10^−3^) are visualized in Fig 4.

To evaluate the statistical significance of the discovered consensus interactions, we used the 10 random sample permutation results from each cohort. We repeated the same procedure described above 100 times, but each time, selecting the results from one of the 10 randomly permuted sample labels from each cohort, and generated consensus p-values for these random results. We applied several cutoffs to the consensus p-values (geometric mean) (5.0 × 10^−5^, 1.0 × 10^−4^, 5.0 × 10^−4^, 1.0 × 10^−3^, 5.0 × 10^−3^, 1.0 × 10^−2^, and 5.0 × 10^−2^) and counted how many of interactions from the real consensus table met the cutoff relative to the permuted results to derived an empirical p-value for the BPM, WPM and PATH consensus observations independently. For BPMs, our analysis suggested the real consensus results were significantly larger than expected at the chosen cutoffs (5.0 × 10^−5^, 1.0 × 10^−4^, 5.0 × 10^−4^) (p < 0.02). For WPM and PATH, we tested geometric mean p-value cutoffs of (1.0 × 10^−3^, 5.0 × 10^−3^, 1.0 × 10^−2^, and 5.0 × 10^−2^). The WPM consensus interaction set was significantly larger than expected by a consensus p-value cutoff of 0.05 (*p* = 0.05). The PATH consensus interaction set was significantly larger than expected by chance (*p* ≤ 0.05) with consensus p-value cutoffs of (1.0 × 10^−3^, 5.0 × 10^−3^, 1.0 × 10^−2^). Detailed information for all consensus interactions is reported in (S5A–S5D Table).

### Analysis of interactions for the glutathione conjugation pathway

From the consensus interaction analysis, we identified the glutathione conjugation pathway as a major source of genetic interactions in multiple cohorts. As a PATH hub interaction, glutathione conjugation was deemed significant in MCS LTN (LTN517) and SBCGS (CHN799) (*FDR* ≤ 0.25) and was also nominally significance in MCS JPN (JPN517). Given this strong support across different cohorts, we further investigated this pathway.

#### Identifying genes contributing to pathway-level statistics

Given the glutathione conjugation pathway’s emergence as an interaction hub, we wanted to determine which genes in the pathway were driving these interactions. To understand this, we performed an interaction degree analysis on glutathione conjugation genes using the MCS LTN (LTN517), SBCGS (CHN799) and MCS JPN (JPN517) cohorts. We first calculated the interaction degree for all glutathione conjugation SNPs and then summarized them at the gene level. To enable comparison across cohorts, we measured a fold enrichment for each gene. More specifically, we first computed the interaction rate (interaction degree divided by total number of SNPs in the network) and then divided it by the background interaction density in each dataset. This fold enrichment was averaged across all SNPs mapping to each gene in cases where there was more than one. This analysis showed that GSTM1, GSTM4, GSTP1, GSTA5’s interactions are 1.5-fold higher than background interactions in all three cohorts. These results are included in Fig 6A.

#### Identifying between-pathway interactions associated with the glutathione conjugation pathway

Although the glutathione conjugation pathway was enriched for interactions across the genome in multiple cohorts (pathway hub model), BridGE was not able to discover between-pathway or within-pathway interactions associated with glutathione conjugation when run independently on each of the three cohorts. To identify which pathways were interacting with glutathione conjugation gene set, we re-ran BridGE on two of the cohorts (MCS LTN517 and SBCGS CHN799-imputed), but only focused on identifying interactions with glutathione conjugation gene set. Specifically, we limited our hypothesis tests to only BPMs that involved glutathione conjugation. This reduced the number of hypotheses test from more than 400k to less than 2k, substantially improving our power to detect interactions with glutathione conjugation. We detected 16 pathways interacting with glutathione conjugation in MCS LTN (LTN517) and 73 in SBCGS (CHN799-imputed) (*FDR* ≤ 0.25), three of which were in common. All glutathione conjugation related between-pathway interactions that are supported by at least one cohort (*FDR* ≤ 0.25) are listed in S10 Table.

## Supporting information

S1 Table BridGE results from BPC3 cohort based on recessive/dominant combined disease model. (A) List of between-pathway interactions discovered in this cohort; (B) List of within-pathway interactions discovered in this cohort; (C) List of pathway hub interactions discovered in this cohort; (D) Corresponding pathway names for Fig 3A.
(XLSX)

S2 Table Replication analysis using BPC3 as discovery cohort and CGEMS as confirmation cohort. (A) List of replicated between-pathway interactions (BPMs); (B) Replication statistics.
(XLSX)

S3 Table Detailed information about interaction between steroid hormone biosynthesis (SHB) and acute myeloid leukemia (AML). (XLSX)

S4 Table BridGE results from CGEMS cohort based on dominant disease model. (A) List of within-pathway interactions discovered in this cohort; (B) List of pathway hub interactions discovered in this cohort.
(XLSX)

S5 Table Consensus summary of BridGE results across 5 different cohorts. (A) List of between-pathway interactions from the consensus analysis; (B) List of within-pathway interactions from the consensus analysis; (C) List of pathway hub interactions from the consensus analysis; (D) Statistical significance results for consensus analysis; (E) Corresponding pathway names for Fig 4.
(XLS)

S6 Table BridGE results from MCS LTN cohort based on dominant disease model. (A) List of pathway hub interactions discovered in this cohort.
(XLSX)

S7 Table BridGE results from SBCGS CHN cohort based on recessive/dominant combined disease model. (A) List of between-pathway interactions discovered in this cohort; (B) List of within-pathway interactions discovered in this cohort; (C) List of pathway hub interactions discovered in this cohort.
(XLSX)

S8 Table BridGE results from MCS JPN cohort based on dominant combined disease model. (A) List of between-pathway interactions discovered in this cohort; (B) List of within-pathway interactions discovered in this cohort; (C) List of pathway hub interactions discovered in this cohort.
(XLSX)

S9 Table BridGE results from SBCGS CHN (imputed) cohort based on recessive/dominant combined disease model. (A) List of pathway hub interactions discovered in this cohort.
(XLSX)

S10 Table Between-pathway interactions involved with glutathione conjugation from SBCGS CHN and MCS LTN cohorts. (XLSX)

S11 Table Comparison between BridGE pathways with GWAS breast cancer SNPs. List of pathways identified by BridGE across 5 different cohorts with FDR≤0.25 and their link to GWAS genes.
(XLSX)
